# Airtightness evaluation of Canadian dwellings and influencing factors based on measured data and predictive models

**DOI:** 10.1177/1420326X221121519

**Published:** 2022-09-19

**Authors:** Maysoun Ismaiel, Maged Gouda, Yong Li, Yuxiang Chen

**Affiliations:** Department of Civil and Environmental Engineering, 3158 University of Alberta, Edmonton, AB, Canada

**Keywords:** Air leakage, airtightness, energy efficiency, canadian dwellings, machine learning, random forest ensemble

## Abstract

The airtightness of buildings has a significant impact on buildings’ energy efficiency, maintenance and occupant comfort. The main goal of this study is to provide an evaluation of the air leakage characteristics of dwellings in different regions in Canada. This study evaluated the key influencing factors on airtightness performance based on a large set of measured data (73,450 dwellings located in Canada with 11 measurement parameters for each). Machine learning models based on multivariate regression (MVR) and Random Forest Ensemble (RFE) were developed to predict the air leakage value. The RFE model, which shows better results than MVR, was used to evaluate the effect of the ageing of buildings. Results showed that the maximum increase in air leakage occurs during the first year after construction – approximately 25%, and then 3.7% in the second year, after which the increase rate becomes insignificant and relatively constant – approximately 0.3% per year. The findings from this study can provide significant information for building designs, building performance simulations and strengthening standards and guidelines policies on indoor environmental quality.

## Introduction

Air leakage can lead to an increase of 30% or more in a home’s heating and cooling costs^1–3^ and can contribute to problems with moisture, noise, dust and entry of pollutants and insects. Reducing air leakage in dwellings is important for improving energy efficiency and thermal comfort.^4^ Failure to achieve airtightness standards could cause damage to the building’s health (e.g. mould and freezing water pipes) and create additional maintenance costs. Table 1 shows a range of air leakage standards for dwellings that exist in different countries.^5^ As shown in the table, for countries with cold climatic conditions, a higher requirement of airtightness is needed to achieve better energy performance.

**Table 1.** Airtightness standards in different countries.

Countries	Standard and category		Maximum air leakage per floor area,* m^3^/(hr.m^2^)	Maximum air change per hour,** ACH_50_
Canada	NECB 2017^9^ (for above-grade envelope area)		2.50	-
Norway^63–65^	TEK7 & 10^63,64^		Net energy demand	-
	TEK7 & 10^63^ TEK17^65^	Energy-saving measures	-	≤3.0
		Net energy demand	-	≤2.5
	TEK17^65^ Floors ≤3	Energy-saving measures	-	≤1.5
		0.5	-	≤0.6
China	Design Standard for Energy Efficiency of Residential Buildings in Severe Cold and Cold Zones (Floors ≥4)^66^		0.5	-
Denmark^67^	Danish Building Regulations (BR10)^68^		≤5.4	-
	Danish Building Regulations (BR15)^69^		≤3.6	-
	Building Class 2020 (BR10)^70^		≤1.8	-
Iceland^71^	Interior temperature (T_i_) ≥ 15°C		5.40	-
	15°C > T_i_ ≥ 5°C		5.40	-
	With electric heating		2.20	-
	Without electric heating		2.20	-
Sweden***^5^	Boverkets Byggregler Regulations (BBR) – Sweden Residential Building^72^		-	1
Finland^30^	The Finnish building code^73^		4	-
Switzerland^74^	The Swedish building code^75^		-	1.8–3.6
Germany	The German energy conservation regulation^76^		-	2.8
Estonian^26^	National energy efficiency policies and measures in Estonia^77^		-	1 or 3****
Belgium^5^	The Energy Performance of Buildings Directive (EPBD)^78^		-	6

*Air leakage m^3^/(hr.m^2^) at 50 Pa represents the airflow through the building envelope at 50 Pa.^79^

**ACH_50_: air change per hour at 50 Pa pressure difference across building envelope.

***Alternative option for code compliance in Sweden.

****1 ACH_50_ for dwellings with balanced mechanical ventilation with heat recovery and 3 ACH_50_ for dwellings with mechanical ventilation.

Canadians demand significant amounts of energy for home heating, cooling and ventilation purposes^6,7^ due to extreme temperatures and the vast landscape. Additionally, the significant increase in the size and number of dwellings has increased the consumption rate of natural resources in Canada over the years.^8^ In response, improving the energy efficiency of Canadian dwellings has become a focus, and changes have been made to the National Building Code of Canada^9,10^ and ASHRAE 90.1^11^ to limit uncontrolled whole-building air leakage rates.^12^ Therefore, the objectives of this research are: (1) investigating the characteristics of air leakages of Canadian dwellings in terms of key variables, to address the effect of each variable considered, and (2) providing a predictive model to estimate the values of air leakage (i.e. air change per hour at 50 Pa pressure difference across building envelope, ACH_50_) using the machine learning approach.

### Literature Review

Air leakage occurs due to the pressure differential across a building’s envelope, which pushes air out of the building through openings and cracks in the building envelope. To have an accurate description and comparable indication of the building’s airtightness, measurements are usually presented at standard pressure differentials and normalized by three commonly used quantities; the total building envelope surface area, floor area or the volume of the building. Normalizing air leakage using the building’s volume is particularly useful when normalizing airflows. The results are usually expressed by air changes per hour (ACH) at the reference pressure of 50 MPa, ACH_50_. This metric is considered to be convenient since the ventilation rates are quoted in air changes per hour and it is the most common metric reported.^13^ Therefore, the discussion in this study is limited to ACH_50_, which is defined below as shown in Equation (1)^14^

(1) $${\text{ACH}}_{50}-\text{Air}\;\text{Change}\;\text{per}\;\text{Hour}\;\text{at}\;50\;\text{Pa}=\frac{{CFM}_{50}\;X\;60\;min}{volume\;of\;the\;house}$$

where CFM_50_ is cubic feet per minute at 50 Pa, and the common formula of ACH_50_ is in imperial units. However, CFM_50_ can be converted to the corresponding standard SI unit m^3^/s by multiplying its value by a factor of 0.000472. The volume of the house is in cubic feet or m^3^ in SI units.

### Air Leakage of Dwellings

Previous studies discussed the general behaviour of air leakage and investigated different factors affecting the air leakage performance of buildings. Natural Research Council in Canada^15^ provided the technical background for future improvements to whole-building airtightness performance and testing procedures for large buildings. The whole-building airtightness performance of different dwellings was assessed and compared with buildings’ performances with values provided in Canadian codes.^10^ The data used were obtained from literature, industry members and the project team, focussing on Canada, the United Kingdom and the United States. A total of 721 different airtightness tests – with information about the height, the number of storeys and age of dwellings – were categorized into four different building types: institutional, commercial, military and multi-unit residential buildings. 66% of the data were for dwellings located in the USA, 18% in the UK and 16% in Canada. The results showed that more recently constructed buildings are generally more airtight. In addition, it was concluded that there was no strong correlation between building height and airtightness. The results showed that the average airtightness value (at 75 Pa) of commercial buildings, institutional buildings, military buildings and multi-unit residential buildings were 12.67 m^3^/hr.m^2^, 9.39 m^3^/hr.m^2^, 3.60 m^3^/hr.m^2^ and 11.12 m^3^/hr.m^2^, respectively. However, the reported data analysis indicated that the measurements of enclosure airtightness did not correspond directly to the actual air leakage. This is because the air leakage rates depend mainly on the pressure difference caused by wind, stack effect and ventilation system. Therefore, this study concluded that many factors could affect air leakage rates, and a specific value for air leakage is not enough to address the actual complexity of infiltration and exfiltration in large dwellings.

Hamlin and Gusdorf^16^ reported that in Canada the airtightness of dwellings was on average 1.23 ACH_50_ for 47 sampled R-2000 houses and 3.06 ACH_50_ for 222 sampled new conventional houses. Stephen^17^ indicated that locations, climate conditions and zones, and different construction techniques have a significant impact on the dwellings studied. In 1997, National Resources Canada^18^ cooperated with Canada Mortgage and Housing Corporation^19^ to investigate 163 new houses and 1811 older houses in Canada.^16^ The study concluded that there was an increasing trend in airtightness: ACH_50_ was 13.7 in houses built before 1921 and 3.1 in the new houses built from 1991 to 1997. A similar increase was observed based on Green Communities Canada’s^20^ database of 3759 houses. The airtightness measured by ACH_50_ ranged from 4.4 for dwellings built in the late ‘90s to an average of 2.8 for dwellings built between 2006 and 2009. Generally, previous studies have shown a significant improvement in the airtightness of dwellings in Canada.

### Factors Influencing Air Leakage from Literature

Several studies have suggested that floor area, age of the building, number of storeys and insulation type are among the significant factors influencing air leakage.^21^ Chan et al.^22^ analyzed the air leakage measurements in 70,000 houses across the USA and related the air leakage measurements to the building size, year built, geographic region and various construction characteristics. The older and smaller houses tend to have higher normalized leakage areas than newer and larger ones.^22^ Another influencing factor is the type of construction. In Finland, a study investigated the average building air-leakage rate of concrete brick houses and timber frame houses. Results showed that the average ACH_50_ for the concrete blockhouses is 2.3 while the average ACH_50_ for the timber-frame house is 3.9.^23^

The effect of the construction type was addressed in Canada by RDH,^15^ which investigated the correlation between the wall construction type and the airtightness by comparing concrete, masonry, steel-frame and wood-frame buildings. The results showed that the minimum airtightness values for the four wall types were the same; however, the average performance was different. The mean airtightness values for wood-frame, concrete, masonry and steel frame buildings were 9.68 m^3^/hr.m^2^, 10.29 m^3^/hr.m^2^, 16.48 m^3^/hr.m^2^ and 19.36 m^3^/hr.m^2^ at 75 Pa, respectively. The results showed that the wood frame buildings were more airtight than the masonry and concrete buildings, while the steel frame buildings were the least airtight. All wall systems were shown to be able to achieve a better airtight performance based on the building type and its complexity. For example, wood frame buildings are always low-rise buildings while the other types of walls may be found in high-rise buildings.

Bomani Khemet et al.^24^ examined the ACH_50_ measurements of over 900,000 single-family homes in Canada.^24^ They consisted of two types: solid masonry and light wood-framed homes. The average airtightness for all measurements was 5.7 ACH_50_. The results showed that newer homes were more airtight. Approximately 3200 light wood-framed, single-family homes from Ontario, all built within the last decade, were analyzed based on blower door test measurements, which were obtained shortly after construction was completed. Results showed a general trend of increasing airtightness in Canadian residential housing on average, but considerable variability in the air leakage rates among provinces and territories.

Different influencing factors were discussed in previous studies and commonly divided into three categories: geometry, technology and materials, and guidance and supervision.^25^ Factors related to the geometry consisted of the number of storeys, envelope area, floor area and volume, which were considered directly linked to infiltration phenomenon.^2,21,26–28^ In contrast, the window and frame area, frame length and the number of penetrations were not adequately evaluated due to the lack of information available regarding these factors.^5,28,29^ Factors related to the technology and materials, consisting of envelope structure, building method and ventilation system, were found to be directly related to the air leakage and thus significant.^21,26,30,31^ Additionally, the dwelling type was deemed significant by Pan et al.^5,32^ Furthermore, guidance and supervision were also concluded to be highly effective in predicting air leakage performance.^5,26,30,32–34^

Identifying factors affecting air leakage is important for developing a predictive model, which can be used to improve the understanding of air leakage in different dwellings. Literature shows the need to develop reliable air leakage predictive models. The models can be significant for researchers to model the air leakage phenomenon, which is related to energy efficiency and performance. Previous research findings were used as a guide in this study to identify the important factors affecting air leakage behaviour.

### Current Research of Air Leakage Prediction Models and Research Gap

Several approaches can be used to model the airtightness of dwellings for predictions. The four main predictive model types used in airtightness estimation are (1) single-component models, (2) building characteristic models, (3) theoretical models and (4) empirical models.^25^ A single-component model^35,36^ helps address the quality of specific detailing, such as window detail or prefabricated panel joints to improve airtightness properties. A building characteristic model is suitable for design use, in which airtightness is determined using a simple mathematical formula. The flow rates are calculated as a product of several coefficients depending on the type of construction. However, such models can be outdated because of the development in construction techniques and the strengthening of building requirements. Moreover, the infiltration phenomenon is not linked to general building characteristics, which could be a limitation for this type of model. A theoretical model is important for understanding complex flow through details. The theoretical model requires the use of Computational Fluid Dynamics (CFD).^37^ Typically, theoretical models are validated by laboratory tests. Finally, an empirical model is dependent on large data sets that are analyzed utilizing statistical or machine learning methods, such as multivariate regression.^2,24,31,35,36,38^

Prignon et al.^25^ presented a literature review discussing the complexity of developing airtightness predictive models in addition to a discussion of the key concepts of infiltration and airtightness, influencing factors and the previous work related to airtightness predictive models. Prignon et al.^25^ also presented previous studies which are based on equations expressing the flow through individual cracks and openings using CFD (Computational Fluid Dynamics). CFD can be precise depending on the theoretical hypothesis. For example, a simple model would consider only one virtual huge crack (which would be the sum of each real crack) while a complex model would consider each crack individually. Younes et al.^37^ discussed the use of CFD, highlighting the fact that they are not suited for practical use and stated that the main CFD drawback is its computation time. Wang et al.^39^ also explained that the use of CFD in practice is often limited by the computation time and calculation power necessary to obtain reliable models.

Chan et al.,^2,22^ Montoya et al.,^21^ Pan,^5^ Fernàndez-Agüera et al.^28^ and Bramiana et al.^31^ presented empirical regression predictive models using several regression analyses on airtightness. In addition, Li et al.^40^ presented several regression methods including ordinary least squares (OLS), stepwise regression, partial least squares (PLS) and nonlinear fitting with independent variable screening methods to establish an airflow coefficient model. The simulation results show that the improvement with the nonlinear fitting was weaker. The previous studies discussing regression predictive models recommended the need for further studies to focus on obtaining more experimental data for building airtightness and concluded that regression models are accurate in describing buildings close to the set used for the analysis. Consequently, they can also be used as predictive tools but are only accurate for buildings close to the data used for the original analysis.

Krstic et al.^41^ tested the neural network approach to predict the airtightness of a building set in Croatia. This suggested methodology was validated by its application to another set of buildings from the Republic of Serbia.^42^ The authors recommended the neural network approach for air leakage estimation due to its strong prediction capabilities. However, validation is still needed.

Literature showed that further work is required to focus on the development of new airtightness predictive models. Also, supervision and workmanship are parameters that are difficult to model. Therefore, there is a need for effective tools to help designers in their decision-making process concerning airtightness.

## Methods and Results

### Data Collection

The data used in this study were collected from the EnerGuide for Houses (EGH) database. This database is an information management tool for tracking residential energy evaluations and measuring the benefits from the energy evaluations delivered across Canada.^43^ During a detailed house energy efficiency evaluation, energy advisors collected house information, which was then used to evaluate the dwellings’ energy consumption and provide recommendations for energy efficiency retrofit. Each dwelling record contains information on the house’s physical characteristics and its energy requirements.

**Figure 1.**
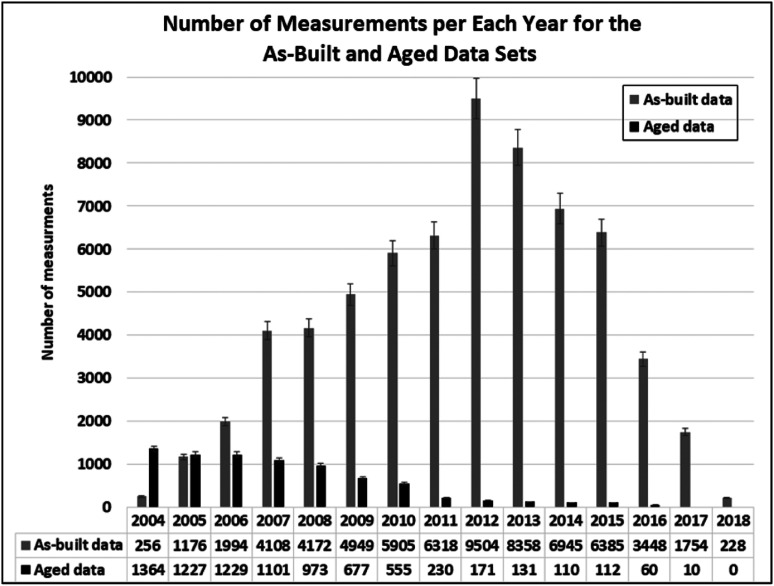
Number of data points per year for aged data and as-built data.

The field data were obtained using the Blower Door Test, which was used to measure the air leakage (in m^3^/s) of a dwelling under a specified pressure difference (e.g. 50 Pa) between indoor and outdoor. ‘Blower Door’ is a device that can pressurize or depressurize a target zone and measure the resultant pressure and airflow through the device.^44^ The technology, first used in Sweden in 1977, involves a fan (blower) mounted onto the frame of an exterior door. The fan pulls air out of the house to lower the internal air pressure, making the pressure outside comparatively higher, and causing air to flow in through all unsealed cracks and openings. The auditors may use a smoke pencil to trace air leaks.^45^ These tests determine the air infiltration rate of a target zone or a building. For each field dwelling measurement, other important characteristics were also recorded: (1) geometric configurations such as the number of storeys, volume and plan shape; (2) thermal characteristics such as the thermal insulation values for the building envelope component, region and age of dwellings; and (3) energy use profiles such as space, heating and ventilation systems. The database used in this study consists of two sets of air leakage data (ACH_50_): 7960 measurements at certain years after construction (denoted as *aged ACH*_*50*_) and 65,500 measurements at the completion of construction (denoted as *as-built ACH*_*50*_) as shown in Figure 1. The aged ACH_50_ measurements were obtained beginning one year after construction to 12 years after construction. Note that *as-built ACH*_*50*_ and *aged ACH*_*50*_ for the same dwellings are not available since air leakages of all the dwellings were measured only once.

**Figure 2.**
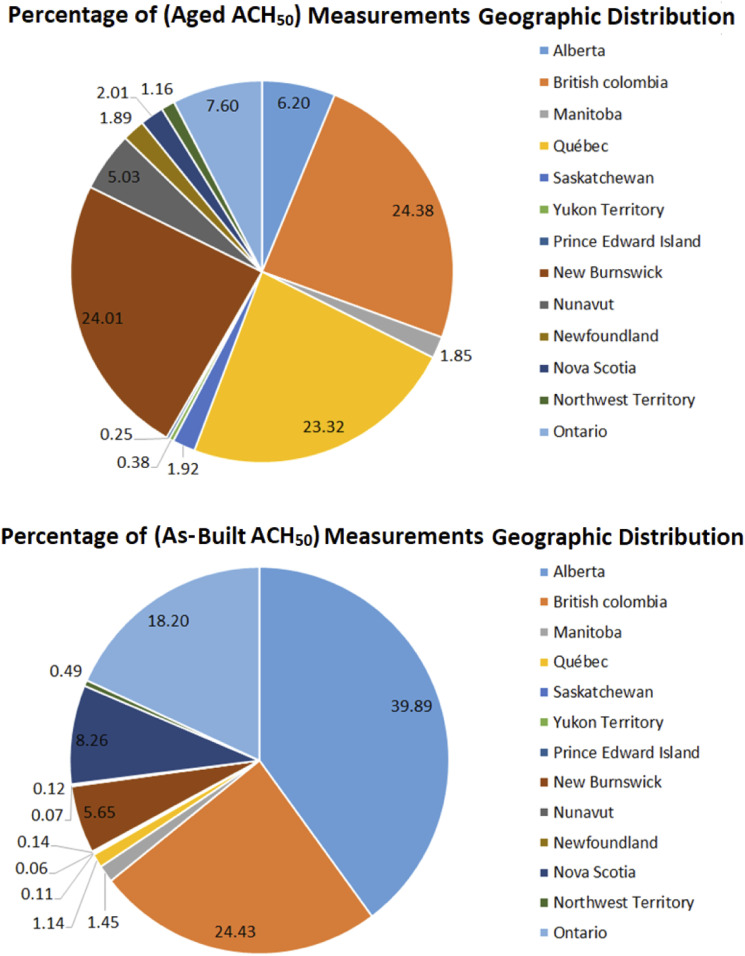
Geographic distribution of the dwellings in the two data sets used in this study.

The distribution of the locations of Canadian buildings for both data sets is shown in Figure 2. The as-built ACH_50_ data set was used to identify the key variables that significantly influence the air leakage directly after construction is completed. All the variables considered in this study for both data sets are summarized in Table 2 with factor levels from the collected data.

**Table 2.** Selected parameters from field measurements.

Variables considered	Factor levels (subcategories involved)
Location (Canada)	10 provinces and 3 territories
	• Single-detached	• Row house end unit
Type of dwelling	• Double/Semi-detached	• Row house middle unit
	• Mobile home	
	• Two-storey	• One and a half
	• One-storey	• Three-storey
Number of storeys	• Split level	• Two and a half
	• Split entry/raised basement	
	• Fans without heat recovery
	• No ventilation system^*^	
Ventilation systems	• Heat recovery ventilator (HRV) • HVI certified HRV^**^ • IMS system^***^
	• Rectangular	• T-shape
Plan shape	• 5–6 corners	• L-shape
	• 7–8 corners	• 9–10 corner
	• 11 or more corners	
Year built	Ranges (2004–2018)
Dwelling volume	Ranges (100–3100 m^3^)
Ceiling insulation RSI value^****^	Ranges (0–20.7)
Main walls insulation RSI value	Ranges (0–10.8)
Foundation insulation RSI value (m^2^K/W)	Ranges (0–9.8)

* No ventilation means that this house has a natural ventilation system by natural forces (e.g. winds and thermal buoyancy force due to indoor and outdoor pressure differences) that drive outdoor air through purpose-built and building envelope openings. Purpose-built openings include windows, doors, solar chimneys and wind towers. This natural ventilation of buildings depends on the climate, building design and human behaviour.

^**^ The Home Ventilating Institute (HVI) is a trade association that recognizes the American Society of Heating, Refrigerating and Air Conditioning Engineers (ASHRAE) Standard 62.2^80^ Ventilation and Acceptable Indoor Air Quality in Low-Rise Residential Buildings as the U.S. ventilation standard.

^***^ The (IMS) is an Integrated Mechanical System for Residential Heating and Ventilation. IMS performance is characterized by two performance descriptors that consolidate measurements for each operating mode to provide annual thermal and electrical performance ratings. The ventilation and water heating loads that are incorporated into the overall ratings are standardized, whereas the space heating loads that are incorporated into the overall ratings are based on the space heating capacity of the individual IMS.^81^

^****^ RSI value (R-value System International, m^2^K/W) is the thermal resistance value that indicates the insulating performance of a material. All RSI values considered in this study are based on construction drawings, not measured.

#### Predictive models

Empirical models are a suitable predictive model type to describe the data and thus are used as the first approach in this study. A multivariate linear regression model was used to relate buildings' airtightness to their building characteristics. The 10-predictor variables were chosen based on the literature review for the multivariate regression analyses: location, year-built type of dwelling, number of storeys, plan shape, volume, ventilation type installed and building enclosure insulation levels such as ceiling, foundation and wall RSI value (R-value System International, m^2^K/W). Multivariate linear regression analyses were performed using a statistical analysis software, SPSS, a predictive analytics software package that facilitates data analysis and model building. Regression was used to relate airtightness measurements and the predictor variables via a general prediction equation. The null hypothesis test was used on the regression parameters in the regression analysis to determine whether each predictor variable was significant and to what extent. The predictor variables were assumed insignificant in the null hypothesis. The significance of each term was evaluated by calculating the *p*-value and comparing it with the significance level chosen. The significance level conventions used in this study are as follows: weak significance ‘between 0.10 and 0.05’, strong significance ‘between 0.05 and 0.025’ and very strong significance ‘less than 0.01’.^46^ The R-square (*R*^2^) is a useful metric to determine the strength of the model. The convention used in this study for *R*^2^ is: weak model strength ‘below 0.3’, moderate model strength ‘between 0.3 and 0.7’ and moderate to strong model strength ‘Above 0.7’.^46^ A multivariate regression model based on the addressed data sets can form a minimum performance target for future airtightness models in both conventional and low-energy homes in this study.

Another machine-learning model was presented in this study using a Random Forest Ensemble (RFE) approach to achieve a better description of the data and improve the model strength. The RFE was used to improve the models' granularity, that is, a manifestation of the level of detail in which the model represents its target system, and the location (i.e. city in this case) variable was also added to the number of independent variables considered. The RFE model showed a high precision in predicting air leakage values ACH_50_. Some limitations should be acknowledged in the data set used in this study. Information about physical characteristics such as the window and frame area, frame length and the number of penetrations were not considered due to the lack of information available regarding these factors. In addition, foundation type, foundation to wall joint type, roofing type, the presence of chimneys and the presence of garages are unknown. Before predictive modelling using multivariate linear regression and random forest ensemble, the univariate descriptive statistics of the dependent and independent (predictor), variables were examined first to explore each variable in the data set, separately. Also, to reveal the ACH_50_ range, and the central tendency of the ACH_50_ values. Generally, univariate descriptive statistics describe the pattern of response to the studied variables and the ACH_50_ trends.

#### Univariate descriptive statistics

**Figure 3.**
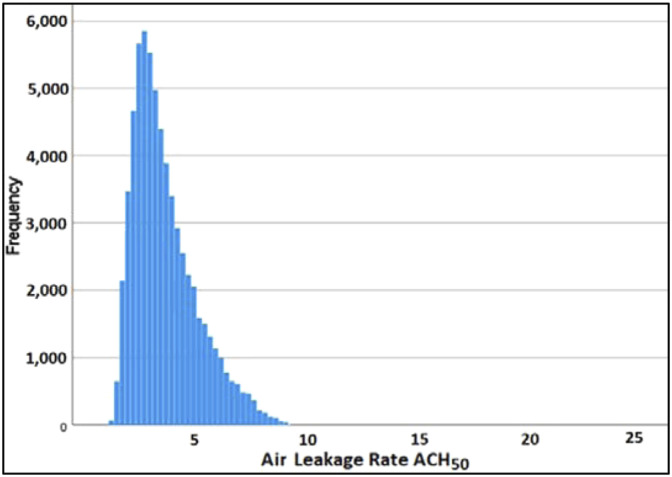
Air leakage rate (ACH_50_) frequency.

A histogram of ACH_50_ in the data set is shown in Figure 3, which indicates an approximately lognormal distribution. Thus, the sample geometric mean^47^ often reflects a more meaningful measure of the central tendency compared to the arithmetic mean due to the exponential decay of the distribution. The geometric mean of ACH_50_ for all buildings in the data set was found to be 2.5, which is smaller than the arithmetic means of 2.86. Similarly, the geometric means of ACH_50_ for the buildings in different provinces and territories are also reported below.

**Figure 4.**
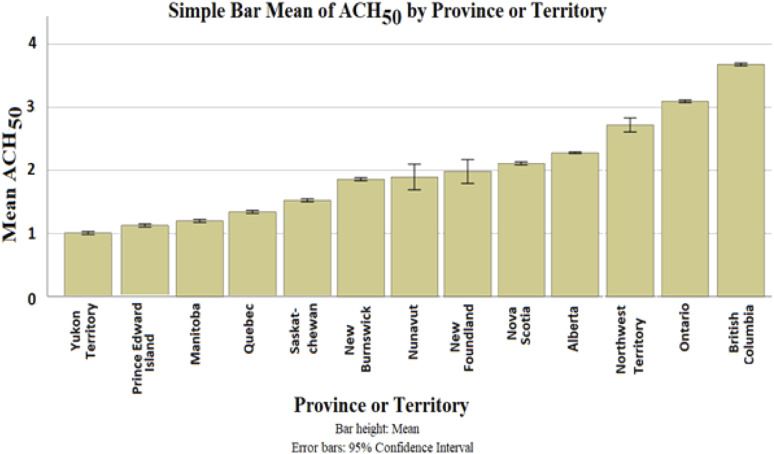
The bar graph with error bars to indicate the variability of data for ACH_50_ in different provinces and territories.

Categorizing the mean air leakage by province, as shown in Figure 4, reveals that Yukon Territory, Prince Edward Island and Manitoba have the lowest air leakage rates based on ACH_50_, while Ontario and British Columbia were shown to have a higher air leakage rate. This may be influenced by climate, construction technique and workmanship.

**Figure 5.**
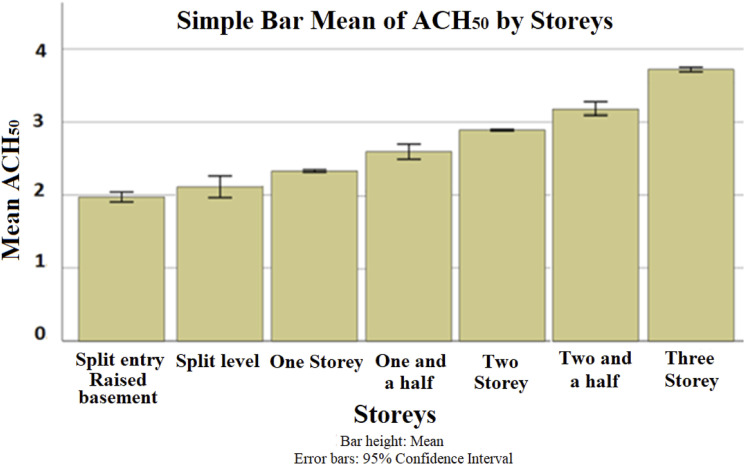
The relation between ACH_50_ and the number of storeys.

**Figure 6.**
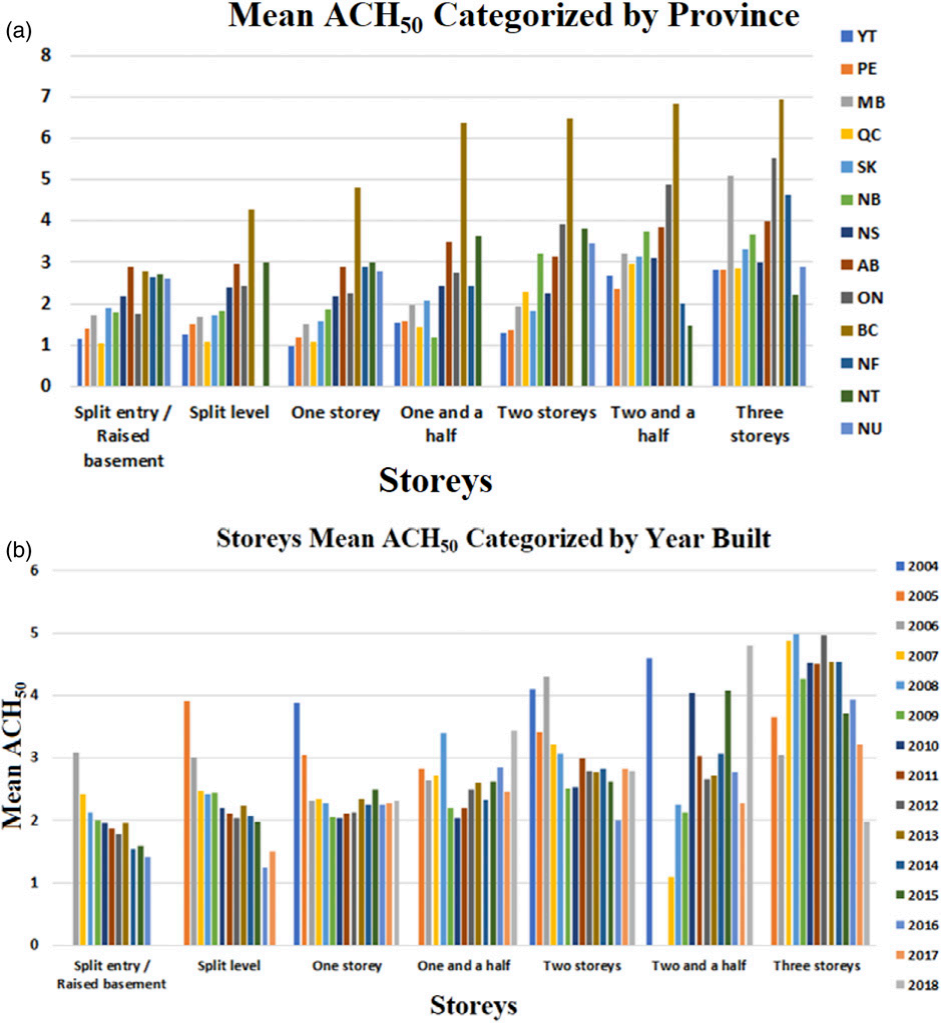
The relation between storeys mean ACH50 categorized by (a) Province and (b) The year built, respectively

An increase by one-storey was found to produce an average increase of 25% in ACH_50_. These lower leakage rates among the one-storey buildings could be due to the reduced housing complexity and the number of leakage joints of single-storey buildings versus multi-storey buildings. Figure 5 shows ACH_50_ categorized by the number of storeys. The association between the parameters was explored and explained by presenting the relationship between variables indicated by the Predictive Measure of Association (Figure 13). The Predictive Measure of Association showed that the number of storeys has a high correlation with the province and the year of building. Figure 6 shows the average storeys ACH_50_ categorized by province (location) and the year of the building, respectively, which shows the same average ACH_50_ trend.

The new dwelling data set exhibits large variation in house volume (i.e. size), with the volume for all dwellings considered in this study ranging from 100 m^3^ to 3100 m^3^. A preliminary analysis was done by considering the average air leakage value of different volume bins (e.g. with a bin size of 100 m^3^). An inversely proportional relationship was observed between the sample mean of ACH_50_ and the volume in all provinces and territories. Therefore, it was concluded that the smaller dwellings tend to have higher ACH_50_ values compared to larger ones. This relationship is likely because the ratio of exterior surface area to volume decreases as the volume increases. Specifically, the air leakage is linearly correlated to the surface area, while the air leakage is normalized by the volume to obtain ACH_50_.

The RSI value of the insulation used for walls, ceilings and foundation was considered in the analysis. The RSI values were not measured but were obtained based on construction drawings. An inversely proportional relationship was indicated between the ACH_50_ and the insulation RSI values. With higher thermal insulation, the envelope experiences less thermal contraction and thereby cracks that could occur due to temperature variation would be smaller.

**Figure 7.**
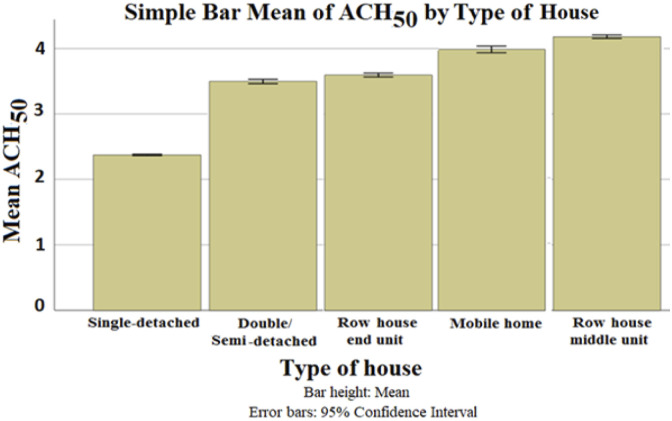
TThe relationship between ACH_50_ and type of house (dwelling)

**Figure 8.**
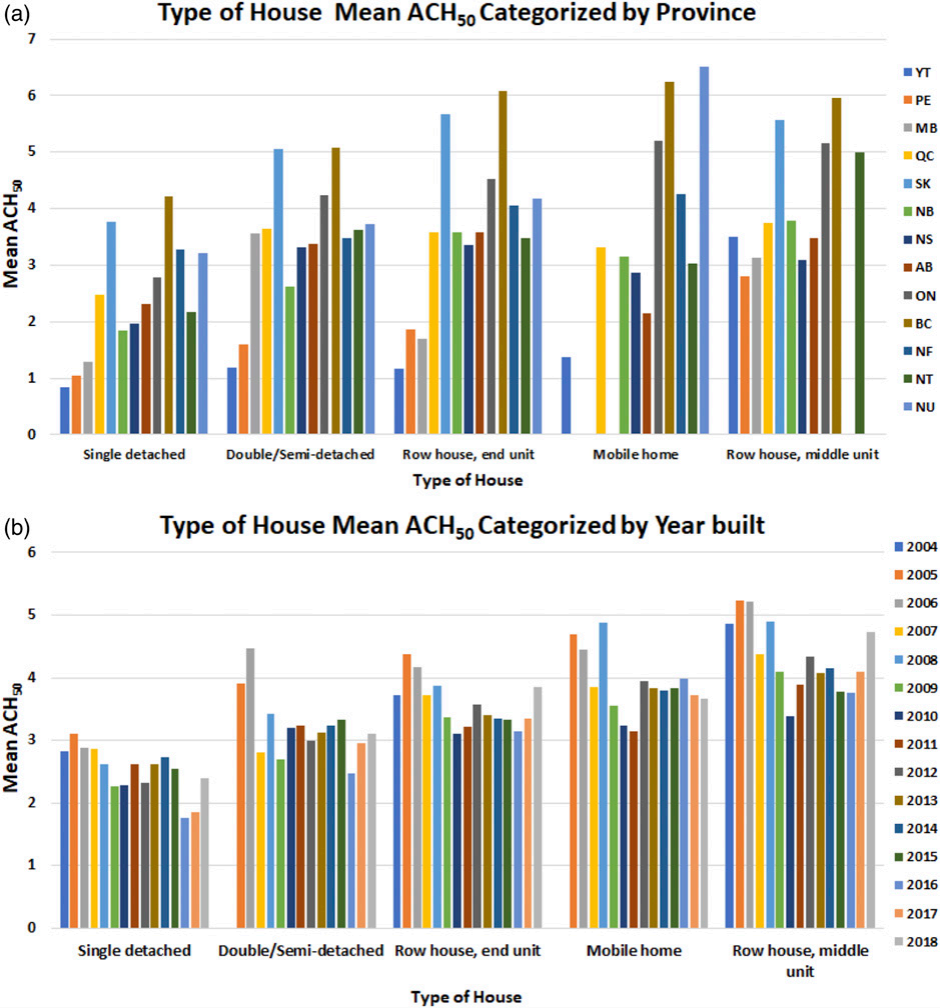
The relationship between ACH_50_ and type of house (dwelling) categorized by (a) province and (b) Year of the building, respectively.

Five dwelling types were considered in this study. The distribution of the addressed data was generally even. Figure 7 shows that the middle units have the highest ACH_50_ value of 4.14 while the lowest ACH_50_ was for the single-detached with 2.3. This also may be influenced by the reduced housing complexity and the number of leakage joints of single-detached buildings. Based on the Predictive Measure of Association (Figure 13), the type of dwellings has shown a high correlation with the province and the year of building. Figure 8 shows the average ACH_50_ of the dwelling type categorized by province (location) and the year of the building, respectively, which shows the same average ACH_50_ trend.

**Figure 9.**
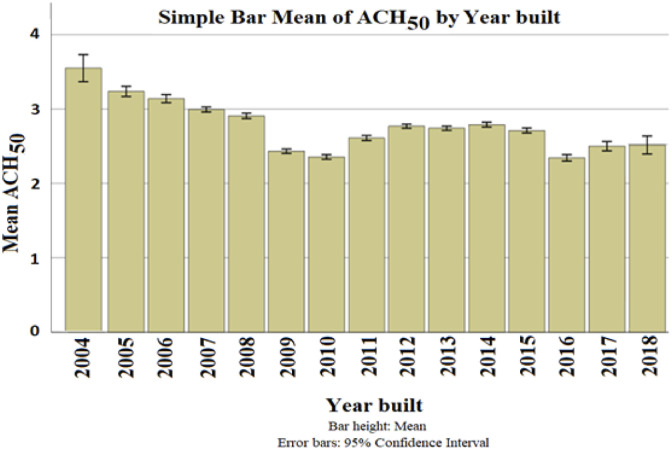
The relationship between ACH_50_ and the year built

**Figure 10.**
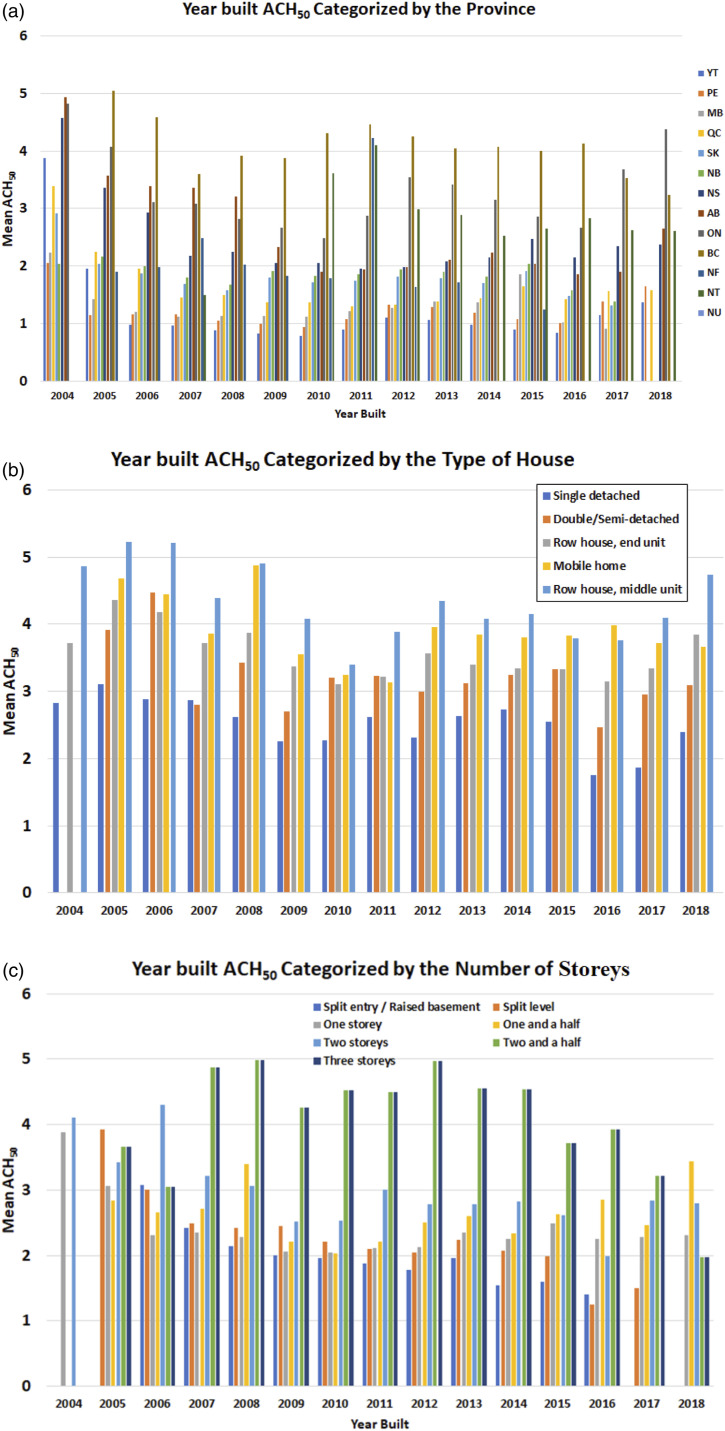
The relationship between ACH_50_ and the year built categorized by (a) Province, (b) Type of house, and (c) Number of storeys, respectively.

Older buildings are expected to have a higher leakage rate due to the rapid change in standards and construction regulations. Technological improvements have resulted from the recent growing awareness regarding energy consumption in buildings. Figure 9 shows a general trend of decrease in ACH_50_ in relation to the year built. From 2004 to 2018, air leakage declined by an average of 35%. This value is for the as-built ACH_50_, which reflects the improvements made to the standards and construction development to achieve a better airtightness performance and reduce energy consumption. A strong correlation was observed between the year built and the type of house, storey number and provinces. Figure 10 shows the average ACH_50_ categorized by province (location), type of house and number of storeys.

**Figure 11.**
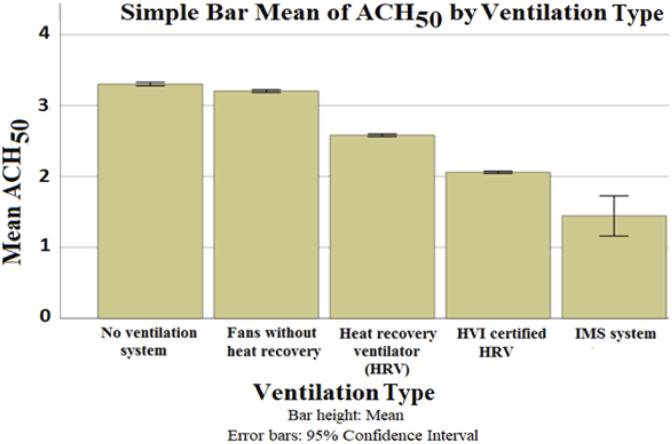
The relationship between ACH_50_ and ventilation systems

Different ventilation types were considered in the data set as indicated in Table 2. In-service infiltration and exfiltration depend heavily on the pressure difference created by wind, stack effect and mechanical ventilation systems.^15^ The effect of the ventilation type on the ACH_50_ is shown in Figure 11. The ventilation type was addressed using the mean ACH_50_ of the addressed data due to the weak correlation between the ventilation type and any other addressed variable as shown by the Predictive Measure of Association (Figure 13). The no ventilation system had the highest ACH_50_. This natural ventilation of buildings depends on the general climate conditions, building design and human behaviour. The IMS (Integrated Mechanical System) has the lowest ACH_50_. The no ventilation system is the only ventilation case that is uncontrolled by mechanical systems, which is the reason for it having the highest ACH_50_ value.

#### Multivariate regression model

Multivariate linear regression was first used to develop a predictive model for ACH_50_. The 10 variables addressed included location, year built, type of dwelling, the number of storeys, plan shape, volume, ventilation system installed and building enclosure insulation levels such as ceiling, foundation and wall RSI. To include categorical variables, such as location and type of dwelling, each non-numerical subcategory for each variable was numerically coded as shown below:

• **Location: **AB=1/ BC=2/ MB=3/ NB=4/ NF=5/ NS=6/ NT=7/ NU=8/ ON=9
• **Type of dwelling:** Single-detached=1/ Row house, end unit=2/ Row house, middle unit=3/ Double or Semi-detached=4/ Mobile home=5
• **Number of storeys:** One-storey=1/ Two-storey=2/ Split level=1.25/ One and a half-split entry=1.5/ Raised basement=1.75/ Three-storey=3/ Two and a half= 2.5
• **Plan shape:** (5–6) corners=6\ Rectangular=4\ (7–8) corners=8\ L-shape=3 \T-shape=1\ 11 or more corners=11\ (9–10) corners=10
• **Ventilation type installed:** No ventilation system=0/ Fans without heat recovery=1/ Heat recovery ventilator (HRV)=2/ HVI certified HRV=3/ IMS system Type 1= 4

Table 3 shows the 10 multiple linear regression coefficients, Standard error, t-statistic, *p*-value and the 95% upper and lower limits. The 10-variable model was based on a sample of over 65,500 dwelling measurements. All coefficients were found to be significant (*p*-value < 0.01). The resultant model for this regression is shown in equation (2)

(2) $$\begin{array}{l} {\text{ACH}}_{50}=\beta_{0}+\left(X_{1}*\beta_{1}\right)+\left(X_{2}*\beta_{2}\right)+\left(X_{3}*\beta_{3}\right)+\left(X_{4}*\beta_{4}\right)+\left(X_{5}*\beta_{5}\right)+\left(X_{6}*\beta_{6}\right)+\left(X_{7}*\beta_{7}\right)+\left(X_{8}*\beta_{8}\right)+\left(X_{9}*\beta_{9}\right)+\left(X_{10}*\beta_{10}\right) \end{array}$$

**Table 3.** Linear regression coefficients 95% CI, (*N* = 65,500).

Input variables	Coefficients	Standard error	*t*-statistic	*p*-value	Lower 95%	Upper 95%
**Intercept**	β_0_ = 42.7182	3.6303	11.7671	6.2081 × 10^−32^	35.60	49.83
**X**_**1:**_ **location**	β1 = −0.0075	0.0018	−4.2228	2.4162 × 10^−5^	−0.01	0.00
**X**_**2:**_ **type of dwelling**	β_2_ = 0.1851	0.0055	33.8260	1.153 × 10^−248^	0.17	0.19
**X**_**3:**_ **number of storeys**	β_3_ = 0.5437	0.0100	54.2602	0	0.52	0.56
**X**_**4:**_ **plan shape**	β_4_ = 0.0099	0.0019	5.3116	1.090 × 10^−7^	0.00	0.01
**X**_**5:**_ **year built**	β_5_ = –0.0191	0.0018	−10.5451	5.6125 × 10^−26^	−0.02	−0.01
**X**_**6:**_ **volume**	β_6_ = −0.0010	0.0000	−47.09	0	0.00	0.00
**X**_**7:**_ **foundation RSI**	β_7_ = −0.2350	0.0049	−48.08	0	−0.24	−0.22
**X**_**8:**_ **wall RSI**	β_8_ = −0.0189	0.0097	−1.94	0.00517	−0.03	0.00
**X**_**9:**_ **ceiling RSI**	β_9_ = −0.1551	0.0046	−33.99	3.6074 × 10^−251^	−0.16	−0.14
**X**_**10:**_ **ventilation type**	β_10_ = −0.3064	0.0055	−55.47	0	−0.31	−0.29

The overall model strength for this regression was moderate on the low side with *R*^2^ = 0.35. Despite the ease of application of the linear multivariate regression model, the model strength must be improved and the non-linear relationship between ACH_50_ and the variables reported in previous studies would need to be considered. In addition, the presence of many non-numerical subcategories in each parameter would demand a better alternative method to describe this data. Therefore, another machine-learning model was used in this study, namely a Random Forest Ensemble (RFE) approach. The RFE was used to improve the models' granularity and the location variable was also added to the number of independent variables addressed.

### Random Forest Ensemble model

There are many types of machine learning algorithms that are often grouped by similarity in terms of their mechanism and approach to analyze data, such as tree-based methods and neural network methods.^48^ Another technique is Ensemble learning wherein multiple models are trained to solve the same problem and are combined as a community model, instead of an individual model, to achieve better results. The main hypothesis is that when constituent models are combined better models can be achieved.^49^ Thus, this study used another machine learning approach, Random Forest Ensemble, which was chosen due to its capability to analyze the data and represent all studied categories in one predictive model.

**Figure 12.**
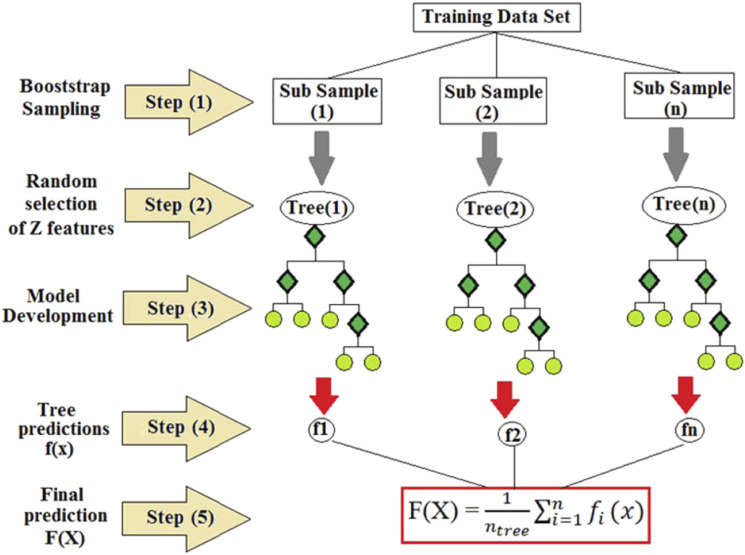
Random forest model process, where: F(x) is the final prediction value, n_tree_ is the number of trees in the forest, f_i_(x) is the prediction result of the i^th^ tree and x is the input vector

The prediction process used in the Random Forest Ensemble learning technique is dependent on several sub-samples; each sub-sample contains a random number of selected readings called trees, which depend on the individual tree structure. The final prediction result was based on a voting mechanism where each decision tree in the forest has a vote, and the final prediction was based on the average of votes as presented in equation (3)^50^

(3) $$F\left(x\right)\frac{1}{n_{tree}}\overset{n}{\underset{i=1}{\sum }}f_{i}\left(x\right)$$

where *F(*x*)* is the final prediction value, *n*_*tree*_ is the number of trees in the forest, *f*_*i*_*(x)* is the prediction result of the *i*^th^ tree and *x* is the input vector. To increase the variability between the decision trees, each decision tree was built from a different subset of the training data set, and the input variables were randomly selected and used to determine each tree split.^51^ In the Random Forest, the bootstrap sampling was implemented; this allows the calculation of the error using the unused subset of the training data set. Figure 12 shows the Random Forest model scheme.

The bagging (bootstrap aggregation) method was considered as the first step in setting up an ensemble learning method. The bagging approach often considers homogeneous weak models and trains them independently from each other in parallel, then combines them following an averaging process.^52^

Depending on the adjusted settings when generating the random forest, each tree continues splitting until there are no more criteria to split (i.e. points had exactly equal values in all criteria) or until it reaches a stopping parameter like a minimum leaf size or a maximum number of splits it could make. The leaf is the end node of a decision tree. The lower the minimum leaf size, the more prone the model is to being overtrained by the training data, which leads to an increase in RMSE when the model is applied to out-of-bag data (validation).^53^ Therefore, training models with different minimum leaf sizes, and then applying them to out-of-bag data proved a good practice for defining the minimum leaf size.

For the suggested RFE model presented in this study, after several minimum leaf size trials, the final minimum leaf size used was 10. Regarding the number of learners used, the literature showed that considering a higher number of learners produced better results.^54–56^ However, it also may lead to overfitting. Therefore, to avoid overfitting, this study trained the model with different numbers of learners and then chose the one after which no more improvement was detected in out-of-bag RMSE. The number of learners used was 100. Random Forests contain a built-in cross-validation method to calculate test set error using out-of-bag samples. Out-of-bag is described as when the training set for the current tree is drawn by sampling with replacement, and a percentage of the cases, usually one-third, is left out of the sample. These excluded cases are called out-of-bag data and are used to get a running unbiased estimate of the classification error as trees are added to the forest variables. These samples were randomly grouped and their effect on the test set error was calibrated, providing one useful method of determining ‘variable importance’.^57^

**Figure 13.**
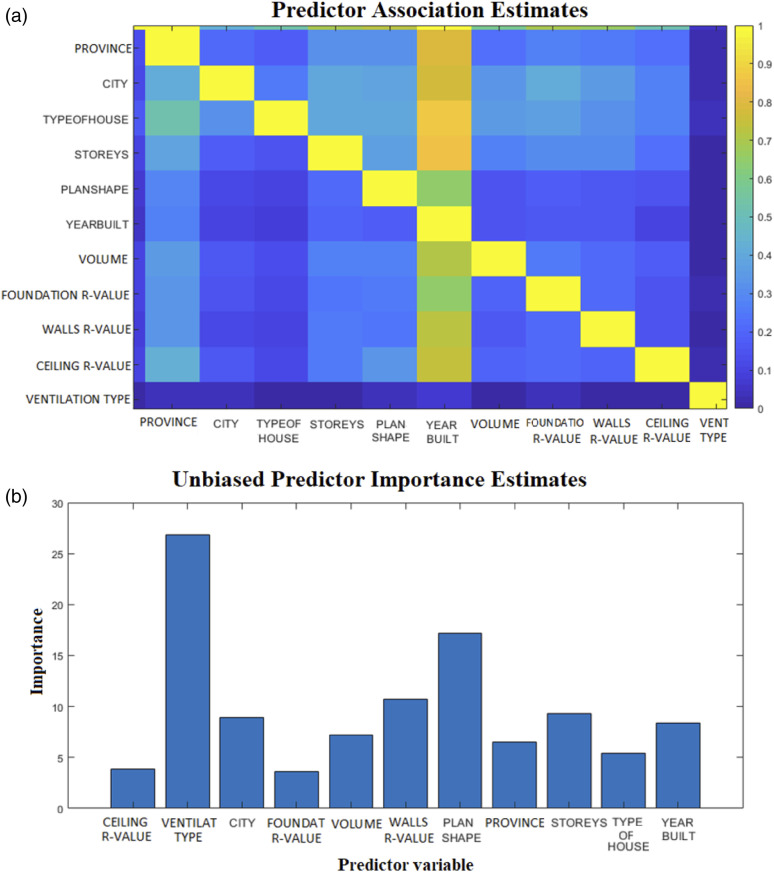
(a) The relationship between variables indicated by the Predictive Measure of Association; (b) Predictor variables comparison using unbiased predictor importance estimation

The prediction process for the ACH_50_ values for different dwellings was suggested by the proposed machine learning model, that is, RFE. To address the relationship between the factors, the Predictive Measure of Association value was obtained. This value indicates the similarity between decision rules that split observations. The strength of the relationship between pairs of predictors can be deduced using the elements of an 11-by-11 matrix of predictor association measures as shown in Figure 13(a). Larger values indicate more highly correlated pairs of predictors.

The approach of rearranging the out-of-bag observations throughout the tree was used to estimate predictors’ relative importance values. This approach averages these parameters over all trees in the ensemble. For each observation that is out of the bag for at least one tree, its weighted mean of the class posterior probabilities was composed by selecting the trees in which the observation was out of the bag. Consequently, the predicted group is the group corresponding to the largest weighted mean. For each observation that is in a bag for all trees, the predicted value is the weighted, most popular value of all the training responses.^58^ Figure 13(b) shows a comparison between the predictors' importance estimates considered in this study. Greater importance estimates indicate predictors that are more important.

To achieve better accuracy, another approach has been proposed to indicate the most important predictors based on the ranking shown in Figure 13(b). The approach used in this study is ‘Recursive feature elimination (RFE)’. RFE aims to find a minimum set of variables, which leads to an accurate prediction model.^59,60^ It started with a Random Forest (RF) built on all variables. The least important variables were then removed, and a new RF was generated using the remaining variables. These steps were recursively applied until a single variable was left. At each iteration, the prediction performance was estimated based on the out-of-bag samples that were not used for model building. The set of variables that leads to the RF with the smallest RMSE was selected.^61^ By applying the REF approach to the studied data set, a slight reduction in the RSME was observed by removing the ceiling insulation RSI value from the studied variables.

**Figure 14.**
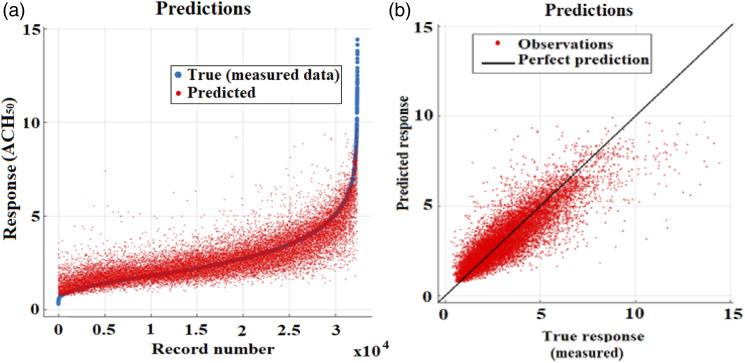
(a) The response of the predicted value versus the actual measurements (true). (b) The predicted response versus measured (true) response.

The measured data were randomly split into training (80%) and validation (20%) subsets, enabling cross-validation.^62^ While the training subset was used for model training, the validation subset was used to test the performance of the trained model on new data. For each variable addressed, further analysis was performed to check both the true response (measured ACH_50_ data) and the predicted response of the currently selected model. This analysis was based on the results from the predictors' importance estimates. The difference between the true (measured ACH_50_ data) and predicted values is shown in the response plot in Figure 14(a), which displays the predicted response versus the record number (number of data). The model showed accuracy for both the validation and trained data sets with an average coefficient of determination (*R*^2^) of 0.79, and root-mean-square error (RMSE) is 0.74, the Coefficient of Variation of the Root Mean Square Error CV(RMSE) of 0.248 was estimated, and Mean Bias Error (MBE) is 0.012. The results showed satisfying consistency between the model’s predictions and the measurements, indicating acceptable predictive capabilities.

To evaluate how well the prediction model performs for different response values, the predicted response versus true response (measured) was plotted. The response of the predicted values to the measured values of ACH_50_ is shown in Figure 14(b) and (a) good correlation between the measured (true) and the predicted value was observed. The plot shows that the model has points scattered roughly symmetrically around the perfect prediction (1:1) line. The vertical distance from the line to any point represents the error of the prediction for that point. As shown by the plot, the majority of predictions are scattered near the line.

The RFE model aimed to predict the ACH_50_ value using the parameters which have a significant effect on the air leakage such as ventilation type, plan shape, insulation, floor, the year of construction, etc. Due to the availability of another data set that provides the air leakage measurements at a certain period after the construction ‘aged ACH_50_ data set’, the authors aimed to investigate the time effect (ageing) from environmental and structural loadings by comparing the ACH_50_ values predicted by the RFE model which provides the ACH_50_ value directly after construction with the measured ACH_50_ values available by the ‘aged ACH_50_ data set’ that provides information on the year the dwelling was constructed and the time when the ACH_50_ was measured (a certain period after the construction; from 1 to 12 years). Due to the availability of two data sets where the age of the building is the main difference, the age of the building was chosen to be investigated further and as an example parameter for analysis in this paper using the REF model and the Aged ACH_50_ data set.

### Influence of Age (Aged ACH_50_)

The age of a dwelling implies two factors: (1) year of construction (i.e. the construction techniques used at the time the dwelling was built), and (2) time (i.e. how long the dwelling has been deteriorating). Due to time effect (ageing) from environmental and structural loadings and factors such as thermal expansion and contraction, material deteriorations, settlement and creep that will cause additional and unintentional cracks, the air leakage rate is expected to increase with time. In this study, the time effect was referred to as the age effect and was separated from the effect of construction technologies. The age effect was evaluated by comparing the data set of aged ACH_50_ obtained from field measurements of existing dwellings to the as-built ACH_50_ values estimated (predicted) by the RFE machine-learning model for the same existing dwellings.

**Figure 15.**
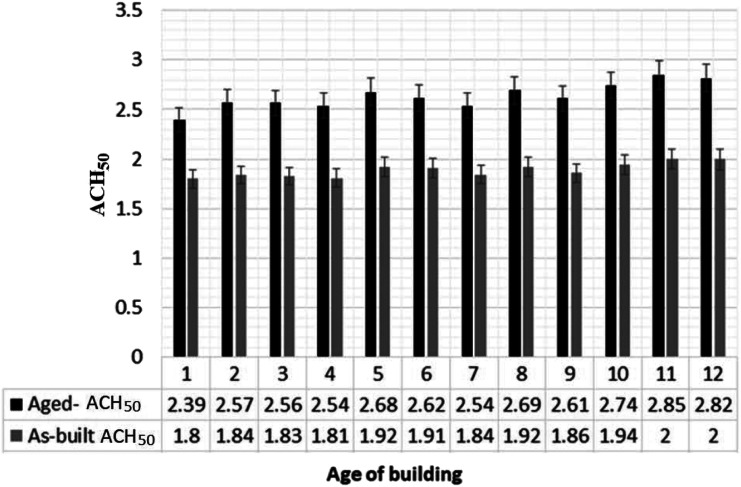
Aged ACH_50_ of existing dwellings at different ages and the predicted as-build ACH_50_ for the same dwellings.

**Figure 16.**
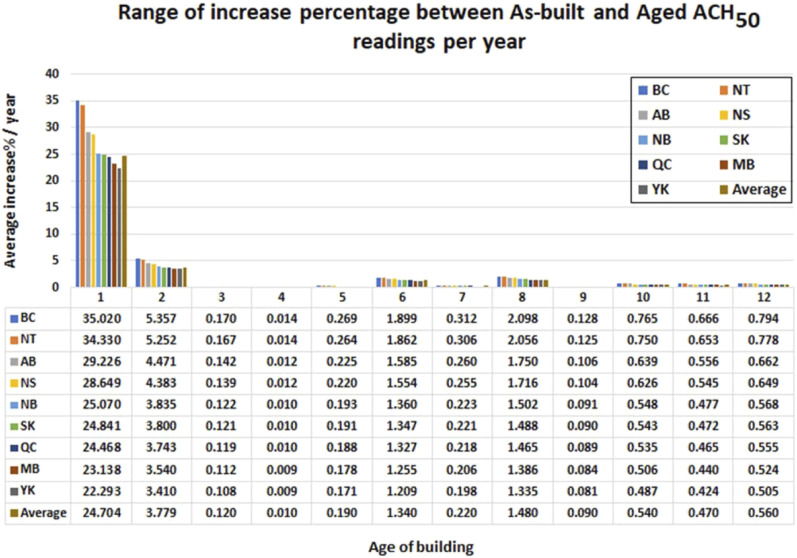
Range of average increase percentage between as-built and aged ACH_50_ readings categorized by location.

The existing dwelling data set (aged ACH_50_ data set) provides information on the year the dwelling was constructed and the time when the ACH_50_ was measured (a certain period after the construction; from 1 to 12 years). The difference between both dates was the age of the dwelling when the ACH_50_ was measured – the aged ACH_50_. The predictive RFE model is capable of estimating the value of the ACH_50_ at the time when the building was constructed – the as-built ACH_50_. Therefore, the age effect on a dwelling can be estimated by comparing its aged ACH_50_ and predicted as-built ACH_50_. In this study, this comparison was conducted for the existing dwellings (Aged ACH_50_) with an age range between 1 and 12 years (i.e. constructed between 2004 and 2017). The year range of the construction was selected to match that of the new dwelling’s data set to make sure that the estimated as-built ACH_50_ was accurate. Figure 15 shows the average as-built and aged ACH_50_ of each age group. Figure 16 shows the range of average increase percentage between as-built and aged ACH_50_ readings per year categorized by location, and Table 4 shows the range of the increased percentages from the as-built ACH_50_ to the aged ACH_50_ (Equation 4) and the average increase percentages per year.

(4) $$\text{Increase}\;\%=100*\left(\text{aged}\;{\text{ACH}}_{50}-\text{as built}\;{\text{ACH}}_{50}\right)/\text{as built}\;{\text{ACH}}_{50}$$

**Table 4.** Range of increase percentage between as-built and aged ACH_50_ readings.

Age of building	Sample size	Increase % between the as-built and aged ACH_50_ to the age of dwellings	
		Average increase %	Average increase % /year
**1**	993	24.71	24.71
**2**	1052	28.49	3.78
**3**	965	28.61	0.12
**4**	977	28.62	0.01
**5**	985	28.43	0.19
**6**	752	27.09	0.34
**7**	644	27.31	0.22
**8**	466	28.79	1.47
**9**	289	28.70	0.09
**10**	257	29.24	0.54
**11**	212	29.71	0.47
**12**	368	29.15	0.56

**Figure 17.**
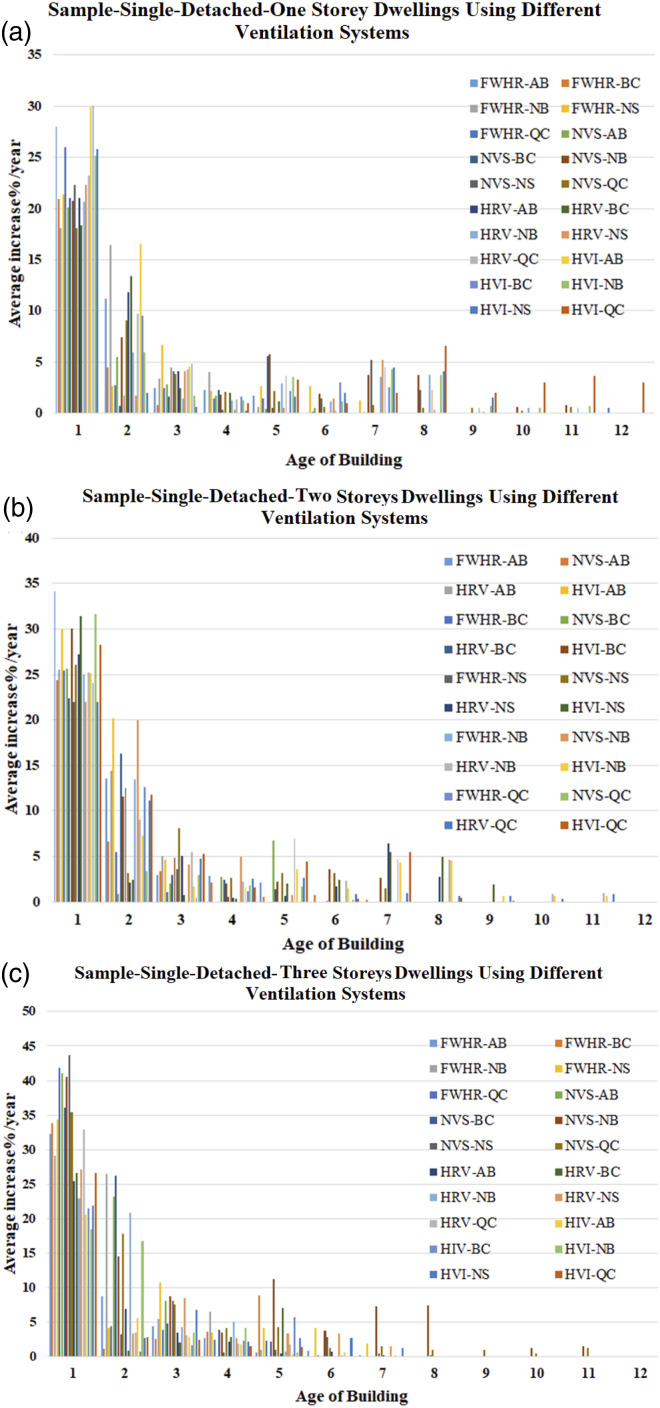
The average ACH_50_ percentages increase per year for single detached dwellings with (a) one storey, (b) two and (c) three storeys, respectively. (FWHR: fans without heat recovery; NVS: no ventilation system; HRV: heat recovery ventilator; HIV: HVI certified HRV).

Samples were constructed to address the average increase of the air leakage percentage per year under the same conditions. For example, Figure 17 shows a sample categorized by province addressing the single-detached type of dwelling, different ventilation types and storeys. These samples were constructed to address the increase in air leakage percentages per year under the same conditions. However, several conditions could be studied further by applying the REF model.

The analysis showed that the average annual ACH_50_ increase for the first year is 25%. This is the maximum increase percentage in the age range considered. Then the second year exceeded the first year by 3.7%. The dwellings’ leakage rate shows a nearly constant average increase rate of 0.3% per year from the third year until the 12^th^ year, which can be assumed for the rest of a building’s life.

## Conclusions and Discussions

The air leakage analysis results from the 65,500 as-built data set of Canadian dwellings studied in this paper have shown improvements in the airtightness of dwellings in Canada from 2004 through 2018 as the ACH_50_ declined by 35%. The airtightness of a dwelling is affected by many factors, some of which have greater effects than others. Based on the studied database, the ventilation type of buildings is an important predictor. A predictive model was introduced using the machine learning approach of Bagging Random Forest Ensemble to predict the ACH_50_ value by considering different variables with multiple subcategories. The predictive model showed an accuracy of an average coefficient of determination *R*^2^ of 0.79 for validation data sets. The root mean square error RMSE was 0.74. Compared to the multivariable regression model with a model strength of *R*^2^ of 0.35, the Bagging Random Forest Ensemble model showed more precise performance in ACH_50_ values prediction. Therefore, the RFEM was used to address the age effect, which was evaluated by comparing the data set of aged ACH_50_ obtained from field measurements of existing dwellings to the as-built ACH_50_ values predicted by the RFE machine-learning model for the same existing dwellings. Results showed that the maximum increase in the ACH_50_ occurred during the first year of the building’s age, when the ACH_50_ was increased by 25% and 3.7% in the second year then the increase rate became small and relatively constant, approximately about 0.3% per year by the third year. The predictive model using the RFE approach presented in this study is limited to simulate data using MATLAB for buildings with the same location ‘Canada’ and climatic conditions as the data used for the original analysis. In addition, the supervision and workmanship factors are not considered in the ACH_50_ simulation. The RFE predictive model can be used to guide future building design in decisions regarding the size, the number of storeys, ventilation type, etc., to achieve better air leakage performance and improve energy efficiency, as well as provide reliable input to building performance simulations. The airtightness predictive model can be significant for the researchers to model the infiltration phenomenon, which is related to energy efficiency and building performance.
